# Engaging patients throughout the health system: A landscape analysis of cold-call policies and recommendations for future policy change

**DOI:** 10.1017/cts.2019.1

**Published:** 2019-05-14

**Authors:** Kelly R. McHugh, Geeta K. Swamy, Adrian F. Hernandez

**Affiliations:** Department of Medicine, Duke University School of Medicine, Durham, NC, USA

**Keywords:** Clinical research, research policy, patient engagement, health system research, research communication

## Abstract

Healthcare institutions may often prohibit “cold-calling” or direct contact with a potential research participant when the person initiating contact is unknown to the patient. This policy aims to maintain patient privacy, but may have unintended consequences as a result of physician gatekeeping. In this review, we discuss recruitment policies at the top academic institutions. We propose an ethical framework for evaluating cold-call policies based on three principles of research ethics. In order to maximize engagement of potential research participants, while maintaining patient privacy and autonomy, we then propose several alternative solutions to restrictive cold-call policies, including opt-in or opt-out platforms, a team-based approach, electronic solutions, and best practices for recruitment. As healthcare has evolved with more collaborative, patient-centered, data-driven care, the engagement of potential research participants should similarly evolve.

## Introduction

It is not uncommon for Institutional Review Boards (IRB) or institutional Human Research Protection Programs (HRPP) to prohibit “cold-calling,” which refers to direct contact with a potential research participant based on prior knowledge of the patient’s health information and in the absence of another (clinical) relationship. The treating physician or other clinical staff are therefore asked to make first contact, and only after gaining physician and patient approval may the research team approach the patient. Although the practice of prohibiting cold calls aims to protect patient privacy, it has unwanted consequences. In this review, we discuss the regulatory landscape of cold-call policies as well as the current state of recruitment policies across top academic institutions. We propose an ethical framework for evaluating the cold-call policies based on three principles of research ethics: autonomy, beneficence, and justice. Within this context, we recommend an approach to recruitment policy that balances patient autonomy and privacy, maximizes patient engagement, and mitigates unnecessary obstacles to research. Specifically, we discuss opt-in and opt-out approaches for future contact, a team-based collaboration between clinicians and researchers, the use of electronic solutions, and a guiding set of best practices. We also bring attention to concerns about privacy, trust, and racial and ethnic disparities in the context of research recruitment, and ways to mitigate these important issues. In light of how healthcare delivery has evolved toward providing more collaborative, patient-centered, and data-driven care, individual IRBs and institutions must reconsider existing policies so that recruitment can similarly evolve.

## Regulations Governing Research Recruitment

In 1991, the Department of Health and Human Services (HHS) published the Federal Policy for the Protection of Human Subjects, or the “Common Rule,” which was adopted by a number of Federal agencies [1]. The Common Rule consists of regulations governing the informed consent process and documentation, IRB responsibilities, and protections for vulnerable populations, founded upon the principles of research ethics identified in the Belmont Report: respect for persons, beneficence, and justice [1,2]. In 1996, the Health Insurance Portability and Accountability Act (HIPAA) was created in order to ease the flow of health information in the electronic era. Under HIPAA, the Privacy Rule established requirements for the exchange, privacy, and security of protected health information (PHI) [3]. Importantly, the Privacy Rule limits disclosure of PHI by covered entities to the minimum necessary to accomplish the purpose of the intended use, without patient authorization [3]. The Common Rule and Privacy Rule are federal research requirements and individual IRBs and Privacy Boards are tasked with creating policies at the institutional level in order to maintain compliance with these rules. However, policies across institutions may vary as a result of differences in the interpretation of these federal regulations. It is within this regulatory framework that policies prohibiting cold-calling were established.

## Cold-Call Policies Across Academic Institutions

The “cold call” refers to contact with a potential research participant by a member of the research team when the patient is unaware that the person initiating contact has knowledge about his or her medical information [4]. In contrast, contact by caregivers is not considered cold-calling, as the patient (potential participant) can be assumed to believe that they are already privy to patient information. Although neither the Common Rule nor the Privacy Rule prohibits cold-calling, many institutions have instituted policies prohibiting the practice in order to protect patient privacy and avoid non-compliance with HIPAA. A review of recruitment policies at 20 institutions with medical schools receiving the highest NIH grant funding in 2018, based on publicly available data, reveals that six institutions make no mention of issues related to cold-calling, twelve have policies advising against it, and two provide guidance for preserving privacy without prohibiting cold-calling (Table 1) [4–23]. Of the twelve institutions with policies that restrict cold-calling, eight specify that cold-calling may be deemed appropriate in certain settings, such as when physician contact is impractical, but this may require a waiver. Most recommend that someone known to the patient introduce the research in person or that an introductory letter signed by the physician be sent to the patient. For patients not receiving care within the hospital’s health system, some institutions require that the primary care physician make first contact [4]. Although these data may not be representative of all academic institutions, grant funding may serve as a proxy for research productivity, and therefore recruitment policies among these institutions are likely to have the greatest impact on research and to set a standard for other institutions.

**Table 1.** Cold-call policies across top academic instructions

Institution^a^	IRB addresses the issue of cold-calling (Y/N)	Cold-calling prohibited (Y/N)	Cold-calling permitted in certain situations (Y/N)	Notes
UCSF	Y	Y	Y	Prospective research subjects should be contacted by someone involved in their care Direct approach by someone not involved in the patient’s care may be approved in exceptional circumstances such as emergency care research or large population-based studies
Johns Hopkins	Y	Y	Y	Researchers can request a partial waiver of the patient’s authorization for recruitment purposes IRB must determine that direct approach by the physician or obtaining the patient’s prior authorization is impractical
Stanford	Y	N	–	Care must be taken to ensure that the potential participant understands how the researcher acquired private information about them, and that the information was obtained in a legitimate manner. If patients are identified via chart review, researcher may consider sending a letter signed by the physician or hospital
Washington University	N	–	–	
Pennsylvania	N			
Pittsburgh	Y	Y	N	IRB prohibits cold-calling of potential research subjects
Columbia	Y	Y	Y	A partial waiver can be granted allowing the university to disclose PHI for the limited purpose of subject recruitment by the investigator
Yale	Y	Y	Y	Approval for direct contact will only be granted when the IRB considers it impracticable for potential participants to be contacted by an individual known to them.
Duke	Y	Y	N	Physician should introduce the study in person or via letter If the patient is not currently receiving care within the health system, permission to contact should be requested from the person’s primary care provider
Michigan	N	–	–	
UCSD	N	–	–	
Mt. Sinai	Y	Y	N	Physician, PCP or clinical team member should introduce study during clinic visit or via letter Physician letters may be used as part of an “opt-out” approach, i.e., researcher can contact the patient if they do not object after receiving the letter
UCLA	Y	Y	Y	When PHI is involved, the patient must initiate contact or agreement to be contacted must be documented by the provider Investigators can contact patients in a recruitment database who provide permission for future contacts
U. Washington	N	–	–	
Northwestern	Y	Y	Y	Exceptions to cold-call policy are made on a case by case basis, i.e., patients who are listed in a registry and agreed to be contacted or are currently in a study with the same investigator
UNC	Y	N	–	IRB discourages cold-calling, but there is no policy against it Extra care should be taken by the researchers and IRB to construct a recruitment approach that respects privacy concerns and anticipates their reaction to contact
Emory	Y	Y	Y	Provider must inform the patient and either provide patient with contact information or get permission to contact the patient Not yet available: patients who sign a front door authorization may be contacted by researcher
NYU	Y	Y	N	Investigator cannot contact subjects identified by the medical record unless they are responsible for the care of that patient
Baylor	N	–	–	
UAB	Y	Y	Y	IRB may permit joint contact by physician and researcher in a letter, or by the researcher referencing the provider in the initial contact Protocols are reviewed on a case by case basis

All information was obtained from publicly available data.

IRB, institutional review board; N, No; NYU, New York University; UAB, University of Alabama at Birmingham; UCLA, University of California Los Angeles; UCSD, University of California San Diego; UCSF, University of California San Francisco; UNC, University of North Carolina; Y, yes.

a Institutions are listed in the descending order of total NIH Grant funding in 2018, according to NIH Research Portfolio Online Reporting Tools (RePORT).

A survey of over 60 institutions in the Clinical and Translational Science Award Consortium about use of the electronic health record (EHR) for patient recruitment adds to these findings. Despite consistent use of the EHR for identifying potential participants and generating recruitment registries, regulatory and recruitment processes significantly varied between institutions. Of note, 53% of institutions required that the primary care provider (PCP) or clinical practice introduce the study when patients were identified via the medical record [24]. It is important to critically evaluate the effects of recruitment policies, especially given the lack of consensus between institutions, so that recruitment strategies best reflect the current needs of patients and researchers. We begin our evaluation with a discussion of physician gatekeeping, a driving force behind the negative consequences of restrictive cold-call policies.

## Gatekeeping

In the context of clinical research, gatekeeping refers to the process by which healthcare providers prevent access to eligible patients for research recruitment [25]. Gatekeeping occurs at both the clinic and patient levels [26]. Physicians may choose to be involved in research based on perceived clinical benefit, relevance of the research question, financial incentives, or potential for academic recognition [26–28]. The decision to approach a patient may be influenced by perceived patient preferences and perceived likelihood of compliance with research [26]. Notably, primary care physicians describe protection from harm as a major motivating factor for gatekeeping, and gatekeeping has been well described in the context of palliative care and pediatric research, where increased patient vulnerability magnifies the protective role of the provider [26, 27, 29]. Sharkey *et al.* have proposed an ethical framework for analyzing gatekeeping related to three principles of research ethics, which we apply to cold-call policies below (Table 2) [25].

**Table 2.** An ethical framework for evaluating policies that prohibit cold-calling

Limitations	
**Autonomy**	
Limits patient choice and control over research involvement	
Physician bias obscures true patient preferences	
Coercion may result from undue influence of the provider	
**Beneficence**	
Under enrollment	
Selection bias	
**Justice**	
Limits potential research benefits for those excluded	
Lack of generalizability of research for groups excluded	
Excess burden on those included	

An ethical framework based on the three principles of research ethics is described.

## Patient Autonomy

Autonomy or respect for human beings refers to respect for a subject’s right to self-determination [2]. When a physician chooses not to approach a patient about research, the patient is denied knowledge and control over his/her full range of research opportunities, and thus the capacity for self-determination is compromised. Although the intent may be protection, the paternalistic action may reflect physician bias that conflicts with patient preferences. Gatekeeping in palliative care research, where low enrollment has notoriously hindered randomized controlled trials, illustrates this concept [30]. Fear of burdening the patient when there is minimal perceived research benefit is a major reason for gatekeeping [27, 31]. However, studies show that palliative care patients often have positive attitudes toward research and derive a sense of self-worth from contributing to future patient care [31]. The disconnect between perceived and actual patient preferences reflects a greater distinction between perceived medical benefits and the broader benefits of research. Medical training promotes a focus on objective measures of physical health, but it is critical to also consider emotional and mental well-being when assessing the value of research. Coercion may also be an issue when the physician is the sole contact allowed. Patients may feel that the decision to participate in research will affect their care or feel personally indebted to their physician. As such, the capacity to make independent decisions may be compromised. Restrictive cold-call policies also have negative implications for the success of research, therefore influencing the next principle of research ethics, research beneficence.

## Research Merit and Beneficence

Beneficence or “do no harm” is another principle of research ethics. Beneficence is defined as a favorable balance of the potential benefits verses potential harms of research [25]. In order to maximize the benefits for the individual and society while minimizing risks, scientific integrity must be ensured [25]. Restrictive cold-call policies have the potential to compromise research integrity by leading to under enrollment and selection bias, thereby exposing some patients to the unnecessary risk of research that may not result in benefits.

The effect of gatekeeping on limiting research enrollment numbers has been described [32]. Requiring action of the busy physician who has multiple responsibilities can cause a delay in recruitment and research completion due to logistics alone [33]. The effects of HIPAA on enrollment have also been recognized. Shortly after the institution of HIPAA in 2003, enrollment universally decreased, causing many to question the Privacy Rule [34, 35]. Under-enrollment is a major reason for trial failure [36]. Beneficence is compromised when trials fail, as enrolled patients are exposed to risks without potential benefits. An analysis of Phase-III clinical trials between 2012 and 2015 showed that 38 clinical trials failed to meet primary or secondary efficacy endpoints. Over 150,000 patients were enrolled, many of whom had cardiovascular disease, diabetes mellitus, or cancer [37]. These high-risk patients were exposed to pharmaceuticals outside of the standard of care, but ultimately did not receive clinical benefit due to trial failure.

By introducing selection bias, policies prohibiting cold-calling also have the potential to compromise research generalizability. We have discussed how physician gatekeeping leads to the exclusion of certain populations. In addition, policies that require patient action to ensure eligibility, such as response to a research invitation, may select for patients who are more functional, motivated, and with fewer comorbidities. This effect has been observed in studies of opt-in vs. opt-out consent [38]. Selection bias is closely related to the ethical principle of justice.

## Justice of Fair Distribution

Justice in the context of research refers to the equitable distribution of both benefits and burdens of research [2]. Gatekeeping effects justice in several ways. First, potentially eligible patients who are excluded are denied the prospect for direct therapeutic benefit from the research. Second, systematic exclusion of certain populations eliminates any potential benefit of the research outcomes for these groups due to lack of generalizability. Finally, one may conclude that the burdens of research are unduly placed on those who are deemed appropriate for recruitment, although the clinical impact of this is unclear [25]. The negative implications of restrictive cold-call policies must be weighed against concerns for patient privacy, which we will discuss in the context of our proposed solutions. First, we consider the changing context of healthcare and research in the United States, which will impact how we evaluate research practices.

## The Changing Landscape of Healthcare Delivery and Research

The central assumption of policies that restrict cold-calling is the importance of the patient-provider relationship. While there is no denying the value of this relationship, the complexity of medicine has necessitated a collaborative approach, in which medicine is delivered via interdisciplinary teams of health professionals. Clinicians collaborate locally to develop institution-specific protocols and nationally to develop evidence-based guidelines. The EHR allows for seamless sharing of patient information. In addition, with increasing scientific advancement and computational power, research and patient care are more closely linked. A learning health system allows for the possibility of continuous analysis of clinical data and integration of knowledge to drive improvements in care [39]. Patients may have access to therapeutic benefits in real time through research enrollment, as opposed to waiting years for intervention approval. Patients are also empowered to take part in their care through the use of wearable devices, mobile health, the Internet, and patient portals (secure online environments connected to the EHR, which allow patients to access their PHI at any time). The use of social media in recruitment is a relevant example of the evolving interplay between technology, healthcare, and patient engagement.

Given the capacity to reach potential subjects, some researchers have successfully utilized Facebook for research advertisement. Through the use of filters (zip codes, gender, age, ethnicity) and interest terms, Facebook allows targeted outreach to certain populations [40–45]. Facebook can also adapt to preferences based on search history. However, these methods are not without concerns. The same technology that allows for tracking patient preferences creates the potential for loss of confidentiality and public exposure of potentially stigmatizing diagnoses or participant status. Communication between research participants can compromise trial design, and lack of face to face contact creates potential for patient misrepresentation [40]. Clearly, healthcare delivery and the interactions between patients and health systems have changed. Research policies must evolve to meet the needs of patients, researchers, and health systems while providing safeguards for patient privacy and research validity. Given this context, in the remainder of this review we propose alternatives to restrictive cold-call policies.

## Proposed Recruitment Policies

There is not one superior approach to participant recruitment, and many areas of uncertainty in research policy during a time of scientific and technologic advancement exist. We do believe, however, that all academic intuitions should have a recruitment policy that specifically addresses the issue of cold-calling, but without strictly prohibiting the practice. Below we provide several recommendations for balancing the obligation to the patient and research, to privacy and autonomy. We discuss opt-in and opt-out approaches, collaboration in healthcare teams, electronic solutions, and best practices for recruitment (Table 3). Central themes include incorporating patient preference, transparency, and patient engagement.

**Table 3.** Recommended alternatives to the restriction of cold-calling: pros and cons

Alternative approach	Pros	Cons
Opt-in/opt-out approaches	Patient preferences are respected Maximizes engagement of those interested in research Preferences are recorded and can be viewed by other researchers	May fail to capture changing preferences or health status Opt-out approaches may compromise autonomy
Collaboration between clinical and research teams	Removes physician gatekeeper Promotes collaboration between clinicians and researchers Provides patients with context for a given research contact	Patient preferences are not included in decisions about research contact Relies on partnership, which may not always be possible
Electronic solutions	Easy access to patient preferences Potential to match patient interests with relevant research studies Broader outreach to patients through portal messages	Potential for security breaches Necessitates training for researchers and clinicians Aggravation from multiple electronic messages
Best practices	Allows for the most flexible recruitment Can be applied to any recruitment policy Recruitment is tailored to the individual patient, diagnosis, and research study	Provides less oversight than a formal policy Greater potential for unfettered recruitment and privacy compromise Patient preferences not part of decisions about research contact

Pros and cons of alternative recruitment models are described.

## Permission For Future Contact: Opt-in and Opt-out

One approach to maximize patient engagement in research is to obtain permission from patients for future contact about research. Institution-wide opt-in or opt-out approaches for future contact are both potential alternatives to restrictive cold-call policies, and successful permission-based platforms, both disease neutral and disease specific, have been described [46–48]. The opt-in approach relies on patient action to ensure eligibility, whereas the opt-out method assumes inclusion unless the patient actively refuses. Both of these options honor patient preferences about research contact, while allowing broader outreach to those who are interested. Patients who wish not to be contacted directly by unknown researchers can still be approached by their physicians. Whether an opt-in or an opt-out approach is most appropriate is up for debate. Some argue that by equating passive acceptance to interest in research contact, opt-out methods compromise patient autonomy [38]. However as mentioned previously, opt-in approaches may have negative implications for enrollment and generalizability, and as maximizing engagement is a key goal in research policy, the benefits of opt-out methods may outweigh the risks [49]. Importantly, opt-in or opt-out permission-based platforms must allow for recruitment decisions to be reassessed and modified, so that those who could benefit from research involvement are not excluded based on prior preferences. This would necessarily require a robust and secure method for tracking and modifying patient preferences. The healthcare team serves as an alternative recruitment model.

## Healthcare Teams

A team-based approach to recruitment relies on collaboration between researchers and clinicians, and may allow for more flexible recruitment practices. A researcher could gain approval for contact from a clinical team, as opposed to an individual clinician. If research teams, such as clinical research units, partnered with clinical teams, contact approval for specific studies may not be necessary. Researchers could then engage patients on behalf of the healthcare team representing a disease process, organ system, risk-based prevention effort, or broad public health effort, thus providing patients with greater context for a given contact. As opposed to opt-in or opt-out approaches, recruitment contacts would be decided by those other than the patient, and thus this method may be viewed as less patient-centered. However, the team-based approach removes the individual physician gatekeeper from recruitment decisions and partners research and clinical care.

## The Electronic Health Record and Electronic Solutions

The EHR provides great potential for storage and sharing of health information for recruitment purposes. Data from the EHR can be deidentified and used for preparatory research activities and then linked back to patients whom researchers wish to contact during research implementation. Alert systems can notify researchers of eligible patients and patient portals can enable delivery of automatic recruitment messages. Permission registries can be built within the EHR or another electronic database. Beyond simply recording research permissions, electronic platforms could be used to store more granular information about research interests and therapeutic priorities. If research opportunities within an institution were also stored electronically, patients could receive notifications about studies of interest, thus maximizing enrollment and therapeutic benefit. The national registry ResearchMatch makes use of such technology [50]. Such platforms could also be expanded to analyze and apply patient preferences to clinical trial design, allowing research studies to be tailored to patient needs. With the institution of new recruitment policies and utilization of innovative technology, it is important to develop best practices to ensure that recruitment remains patient-centered.

## Recommended Best Practices

Institutions may choose not to adopt a specific policy around cold-calling and instead rely solely upon a set of best practice guidelines for patient recruitment. Although this may allow for the most flexible recruitment practices, the relative lack of oversight may be a concern for both IRBs and patients and does not consider patient preference for contact. We recommend that institutions provide best practice guidelines, in addition to a recruitment policy, to ensure that recruitment practices respect privacy. Below we outline our recommendations for best practices.

Recruitment plans must be constructed on a study-by-study basis. For higher risk studies, contacts might be tailored to those meeting strict criteria, thus targeting a specific population who is more likely to be eligible and interested in the research. For minimal risk studies, contacts may be geared to a broader patient population. Patients must always be given sufficient time to consider participation, and the amount of time may be greater for studies with greater risk. A written letter or message via the secure patient portal within the EHR may be preferred as the first mode of contact to allow patients time for independent thought. Extra care should be taken when recruiting those with stigmatizing or distressing diagnoses, such as ensuring that the patient is aware of his or her condition prior to contact, and that the recruitment setting respects privacy. Patients should be informed about how they were identified and provided with the research team’s contact information.

When patients are eligible for multiple studies, it may be necessary for a gatekeeper to guide recruitment prioritization. Instead of the treating physician who may not be fully aware of research participation options, a representative from the relevant department with comprehensive knowledge of the research studies as well as patient specific considerations could provide insight. In some cases, where collaboration is necessary for maximal recruitment, discussion among the interdisciplinary team would be more appropriate to determine eligibility, availability, and potential benefits, such as when relying on an oncology tumor board or when recruiting surgical patients for surgical and anesthesia studies.

Collaboration between researchers and healthcare providers should be encouraged. Clinicians have unique perspective about their patient’s needs and will likely field patient questions. If possible, clinician input should be incorporated into research design and feedback requested throughout the research process. Although they will not serve as gatekeepers to patient recruitment, as an essential part of the healthcare team, when possible, physicians should be notified about enrollment decisions influencing the medical care of their patients. When considering recruitment policies and best practices, several issues related to cold-calling deserve independent discussion.

## Privacy

In line with HIPAA, policies that restrict cold-calling aim to protect patient privacy and confidentiality by limiting disclosure of PHI. Although a greater number of people may have access to patient information when other practices are substituted for a policy prohibiting cold-calling, recruitment efforts should not be unfettered. The IRB or Privacy Board must evaluate a researcher’s study specific plans for recruitment, and safeguards for ensuring protection and minimum necessary access to PHI must be approved by those committees. Training for research coordinators about privacy protection and published templates for letters, phone scripts, etc., could ensure standardized practices. In addition, electronic recruitment methods should utilize privacy safeguards, such as firewalls, encryption, and authentication [51].

Restricting cold calls also prevents against patient aggravation from multiple research contacts. However, as described, recruitment plans aligning with best practices would be tailored to both study and patient to prevent research invitations becoming equivalent to spam. Through the use of electronic platforms, patient feedback about future contact could be recorded and updated as needed and improved over time. Central documentation of research enrollment and evaluation of patient interest level could also allow for assessment of future contacts. Guidelines for limiting communication could be modeled after studies that have utilized successful phone surveys [52].

## Trust

Patient trust is a central determinant of research participation. When cold-calling is prohibited, necessitating involvement of the healthcare provider in research recruitment, patients may have greater trust in research due to inherent trust in the provider [53]. Transparency and engagement are necessary to build similar confidence in the research community. Lack of trust in research may largely be related to lack of knowledge. Studies have shown that patients are generally unaware of how their information is used and the safeguards in place to prevent abuses [53]. Surveys also show that patients feel negatively about private sector involvement and use of data for profit, but they feel positively about the societal benefits of research [53]. During recruitment, these issues should be addressed through language that is accessible in order to alleviate any patient concerns about misuse of data. Returning research results to participants is another way to enhance trust and create a sense of partnership between participants and the research community.

In accordance with Section 801 of the FDA Amendments Act and the Final Rule for Clinical Trials Registration and Results Information Submission (42 CFR part 11), clinical trials that meet certain criteria are required to register and report summary results on clinicaltrials.gov [54]. A complementary NIH policy specifies that all NIH-funded clinical trials register and report results on clinicaltrials.gov [55]. However, these reports are generally not written in lay language, and are not disseminated specifically to research participants [56]. While the FDA and HHS are silent on the topic, the Secretary’s Advisory Committee on Human Research Protections (SACHRP) has provided recommendations for return of results to participants directly. The SACHRP promotes return of both general and individual results, with emphasis on results that are valid and actionable [56, 57]. They do, however, recognize that return of results is not without potential consequences. For example, if participants confuse research results with clinical results, they may take inappropriate medical action based on findings lacking clinical significance. Thus, ethical considerations regarding return of results must be considered on a case by case basis. When investigators do not plan to share results, due to logistical concerns, potential for distress, uncertain clinical significance, etc., this should be addressed in the informed consent so that participants are aware of this at the outset.

Patient engagement will also help build trust. Outreach in the form of newsletters or flyers with research results could promote awareness and a sense of belonging to the larger research effort. By soliciting patient preferences, electronic methods could also allow patients to take a more active role in the research process. When the public feels that its voice is heard, a true partnership between subjects and researchers will be formed. Trust is particularly a concern for minority populations.

## Racial and Ethnic Disparities

Racial and ethnic minorities are underrepresented in research [58]. The reasons are multifactorial, and include socioeconomic disparities, language barriers, and mistrust arising from historical harms. Removing the provider from research recruitment may preferentially diminish racial/ethnic minority involvement. Studies have shown that disparities in patient portal use among racial and ethnic minorities are in part due to a greater value placed on in-person communication with providers by the minority population, in order to ensure the highest quality of care [59–61]. Transparency and engagement are especially critical for reaching these groups. Language barriers are another concern. Current recruitment strategies may fail to capture non-English speakers, due to lack of bilingual staff. Although most patient portals utilize English only, several health networks now utilize Spanish-language portals. These include Spanish versions of the patient portal MyChart, known as MiRecord and MiSalud [62–64]. Universal adoption of multilingual portals may help improve outreach to non-English speakers in ways that traditional recruitment methods have not.

## Limitations

There are several limitations of this review. Our analysis of recruitment practices across academic institutions was based on publicly available data. It is possible that these data may not accurately reflect true recruitment practices. For those institutions listed as not addressing cold-calling, it may be that there are unwritten rules for handling recruitment that are engrained in the culture of the institution. We did not contact IRB officials or representatives of the institutions directly in order to further explore these policies. However, the degree to which recruitment policies are displayed publicly for researchers and others to access may reflect the degree to which they are enforced. Additionally, we surveyed the top 20 institutions with the highest NIH grant funding in 2018, and thus the sample may not represent the entire research community. However, as noted, these institutions are likely to have the most impact on research and therefore are worthy of discussion.

## Conclusions and Future Directions

As technology continues to expand, enabling new methods of healthcare engagement, communication, and novel therapeutic advances, research policy must also adapt to maximize benefits for patients and society. Although the restriction of cold-calling aims to promote patient privacy, given the complexity and collaborative nature of medicine, the individual provider may not have sufficient information or resources to facilitate recruitment decisions. We call for institutions to abandon strict cold-call policies, and adopt recruitment strategies that balance patient choice, privacy, and research success. Institution-wide opt-in or opt-out platforms offer the benefit of honoring patient preferences, while enabling broader engagement. Collaboration between researchers and clinicians in teams may allow for flexible recruitment that aligns research and clinical care. The EHR and electronic solutions offer potential to personalize recruitment and encourage patient feedback. Regardless of the policy instituted, best practices that reflect a commitment to privacy and transparency must be established and will help promote a culture of research engagement.

Many questions, both ethical and technical, regarding recruitment policies exist. It is not clear how providers and patients will perceive use of the EHR for research. With experience and feedback, necessary changes can be made to increase efficiency and usability of the EHR for research purposes. In addition, without directly involving the treating physician in recruitment decisions, it is possible that a greater portion of ineligible patients will be approached. The overall effect of eliminating the gatekeeper on the efficiency of the recruitment process must be assessed. Providers may be increasingly faced with questions from patients about research studies, and may not have the necessary information to address these questions. The research community will need to address any concerns of providers and ensure that patients have contacts in order to obtain more information about research opportunities. The number of minority patients utilizing patient portals may increase with the adoption of multilingual portals, which may or may not allow for greater minority involvement in research. As institutions adapt and implement new policies, we will continue to gain clarity about these uncertainties.
